# Comparison of Immunity in Mice Cured of Primary/Metastatic Growth of EMT6 or 4THM Breast Cancer by Chemotherapy or Immunotherapy

**DOI:** 10.1371/journal.pone.0113597

**Published:** 2014-11-19

**Authors:** Reginald M. Gorczynski, Zhiqi Chen, Nuray Erin, Ismat Khatri, Anna Podnos

**Affiliations:** 1 University Health Network, Toronto General Hospital, Toronto, Canada; 2 Department of Immunology, Faculty of Medicine, University of Toronto, and Institute of Medical Science, University of Toronto, Toronto, Ontario, Canada; 3 Department of Medical Pharmacology, Akdeniz University, School of Medicine, Antalya, Turkey; Istituto Superiore di Sanità, Italy

## Abstract

**Purpose:**

We have compared cure from local/metastatic tumor growth in BALB/c mice receiving EMT6 or the poorly immunogenic, highly metastatic 4THM, breast cancer cells following manipulation of immunosuppressive CD200:CD200R interactions or conventional chemotherapy.

**Methods:**

We reported previously that EMT6 tumors are cured in CD200R1KO mice following surgical resection and immunization with irradiated EMT6 cells and CpG oligodeoxynucleotide (CpG), while wild-type (WT) animals developed pulmonary and liver metastases within 30 days of surgery. We report growth and metastasis of both EMT6 and a highly metastatic 4THM tumor in WT mice receiving iv infusions of Fab anti-CD200R1 along with CpG/tumor cell immunization. Metastasis was followed both macroscopically (lung/liver nodules) and microscopically by cloning tumor cells at limiting dilution in vitro from draining lymph nodes (DLN) harvested at surgery. We compared these results with local/metastatic tumor growth in mice receiving 4 courses of combination treatment with anti-VEGF and paclitaxel.

**Results:**

In WT mice receiving Fab anti-CD200R, no tumor cells are detectable following immunotherapy, and CD4+ cells produced increased TNFα/IL-2/IFNγ on stimulation with EMT6 in vitro. No long-term cure was seen following surgery/immunotherapy of 4THM, with both microscopic (tumors in DLN at limiting dilution) and macroscopic metastases present within 14 d of surgery. Chemotherapy attenuated growth/metastases in 4THM tumor-bearers and produced a decline in lung/liver metastases, with no detectable DLN metastases in EMT6 tumor-bearing mice-these latter mice nevertheless showed no significantly increased cytokine production after restimulation with EMT6 in vitro. EMT6 mice receiving immunotherapy were resistant to subsequent re-challenge with EMT6 tumor cells, but not those receiving curative chemotherapy. Anti-CD4 treatment caused tumor recurrence after immunotherapy, but produced no apparent effect in either EMT6 or 4THM tumor bearers after chemotherapy treatment.

**Conclusion:**

Immunotherapy, but not chemotherapy, enhances CD4^+^ immunity and affords long-term control of breast cancer growth and resistance to new tumor foci.

## Introduction

The immunoregulatory molecule CD200 has been reported to regulate growth of human solid tumors [1], [2] and hematological tumors [3]–[5]. Using a transplantable EMT6 mouse breast cancer line CD200 expression, by tumor cells or host, increased local tumor growth and metastasis to DLN [6], [7], which was abolished by neutralizing antibody to CD200, or following growth in mice lacking the primary inhibitory receptor for CD200 (CD200R1KO mice). In contrast to these observations, growth of the highly metastatic 4THM breast tumor (derived from a 4T1 parent line) was increased in CD200R1KO mice, with somewhat diminished growth in CD200^tg^ animals [8].Surgical resection in CD200R1KO EMT6 tumor-bearing mice, followed by immunization with CpG as adjuvant, cured CD200R1KO mice of breast cancer recurrence in the absence of lung/liver metastases, and of micro metastases (defined by limiting dilution cloning in vitro) in DLN [9].

Multiple factors both intrinsic to tumor cells themselves and host associated elements are implicated in tumor metastasis [10]–[14]. Many such factors are associated with altering trafficking of either host inflammatory-type cells to the local tumor environment where they can facilitate metastasis through a variety of mechanisms [15]–[17], including regulation of host resistance mechanisms [18]–[21]. Metastatic tumor cells are known to undergo changes in gene expression profile leading to increased cancer stem cell- like properties and the ability to survive, establish and grow in a foreign environment [22]–[24]. Like CD200, an inhibitory member of the B7 family of T cell co stimulation, expression of another such molecule, B7× (B7-H4) has been reported to influence metastasis using 4T1 tumor cells and B7KO mice [25]. B7KO mice with 4T1 tumors, like CD200R1KO with EMT6, showed enhanced survival and a memory response to tumor re-challenge, which was correlated with decreased infiltration of immunosuppressive cells, including tumor-associated neutrophils, macrophages, and regulatory T cells, into tumor-bearing metastatic lung tissue [25]. CD200R1KO mice showed increased growth of 4THM tumors [24].

The studies below compared protection seen in surgically treated/immunized EMT6 or 4THM tumor injected WT mice with/without manipulation of CD200:CD200R interactions using Fab anti-CD200R, with attenuation of disease after surgical resection followed by chemotherapy.

## Materials and Methods [9]

### Ethics approval and animal use guidelines

This study was carried out in strict accordance with the recommendations of the Canadian council for Animal Care (CCAC). The protocol was approved by the Committee on the Ethical use of Animals for experimentation at the University Health Network (Permit Number:AUP.1.5). All surgery was performed under sodium pentobarbital anesthesia, and all efforts were made to minimize suffering.

### Mice

CD200KO and CD200R1 knockout mice are described elsewhere [9]. WT BALB/c mice were from Jax Labs. All mice were housed 5/cage in an accredited facility at UHN. Female mice were used at 8 wk of age.

### Monoclonal antibodies, and CpG deoxyoligonucleotide for adjuvant use, are described elsewhere [6], [9], [26]

Rabbit Fab anti-CD200R1 antibody was prepared using a commercial kit (Pierce Protein Products, Rockford, IL, USA) and rabbit IgG isolated by Cedarlane Labs (Hornby, Ontario, Canada), following immunization of rabbits with 500 µg mouse CD200R1 emulsified in Freund's Adjuvant. In independent studies (not shown) this antibody (1∶1000 dilution) inhibited binding (FACS analysis) of FITC-labeled mouse CD200 to Hek cells transduced to over-express murine CD200R1.

### EMT6 breast tumor cells, induction of tumor growth in BALB/c mice, and limiting dilution cultures to establish frequency of metastasis to draining lymph nodes (DLN) were as described earlier [9], [26]

4THM tumors, a highly metastatic variant of 4T1, were derived by Erin et al as reported elsewhere [24].

### Surgical resection and immunotherapy/chemotherapy of tumor-bearing mice [9]

Mice receiving 5×10^5^ EMT6 or 1×10^5^ 4THM tumor cells injected into the mammary fat pad in 100 µl PBS underwent surgical resection 14–16 d later. For immunotherapy, mice received intraperitoneal immunization with 3×10^6^ EMT6 (or 4THM) tumor cells (irradiated with 2500Rads) mixed with 100 ug CpG ODN (see above) in 100 µl PBS, emulsified with an equal volume of Incomplete Freund's adjuvant, 2 days after surgery. Mice treated with chemotherapy post surgical resection, received 4 injections of paclitaxil intraperitoneally in 0.15 ml PBS (Taxol: 10 mg/Kg), beginning on the day of surgery, and at 21 day intervals thereafter. In addition, beginning on the day following surgery, and at 14 day intervals for a total of 6 injections, the same mice also received anti-VEGF (30 mg/Kg) iv in 0.3 ml PBS.

All animals were monitored ×3/week for weight loss and general health and sacrificed at the times indicated in individual experiments (>10% weight loss), with visible tumor colonies in the lung/liver enumerated. DLN cell suspensions were prepared from individual mice and cloned under limiting dilution in 96-well flat-bottomed microtitre plates to assess tumor colony formation [7]. Important variables measured were time post treatment to sacrifice, and tumor growth-note that aggressive uncontrolled tumor growth in some groups in individual experiments led to certain groups being sacrificed before others (see text).

### Preparation of cells and cytotoxicity, proliferation and cytokine assays: see [9], [26]

In brief, 5×10^6^ splenocytes from mice treated as described in the text were stimulated in vitro in triplicate with 2×10^5^ irradiated (2500Rads) tumor cells in 2 ml αMEM with 10% fetal calf serum. 100 µl aliquots of supernatants were assayed at 48 hr for various cytokines using commercial kits (BioLegend, San Diego, USA). Cells were harvested from cultures at 6 d, washed ×2, and incubated for 18 hr with 1×10^3^ ^3^HTdR-labelled tumor target cells at varying effector:target ratios to determine direct anti-tumor cytotoxicity.

### Statistics

Cloneable tumor cell frequency was determined as before [6]. Within experiments, comparison between groups used ANOVA, with subsequent paired Student's t-tests as indicated.

## Results

### Surgical resection followed by immunization along with Fab anti-CD200R, or chemotherapy alone, prevents metastasis of EMT6, but not 4THM, in BALB/c mice

**Figure 1:**
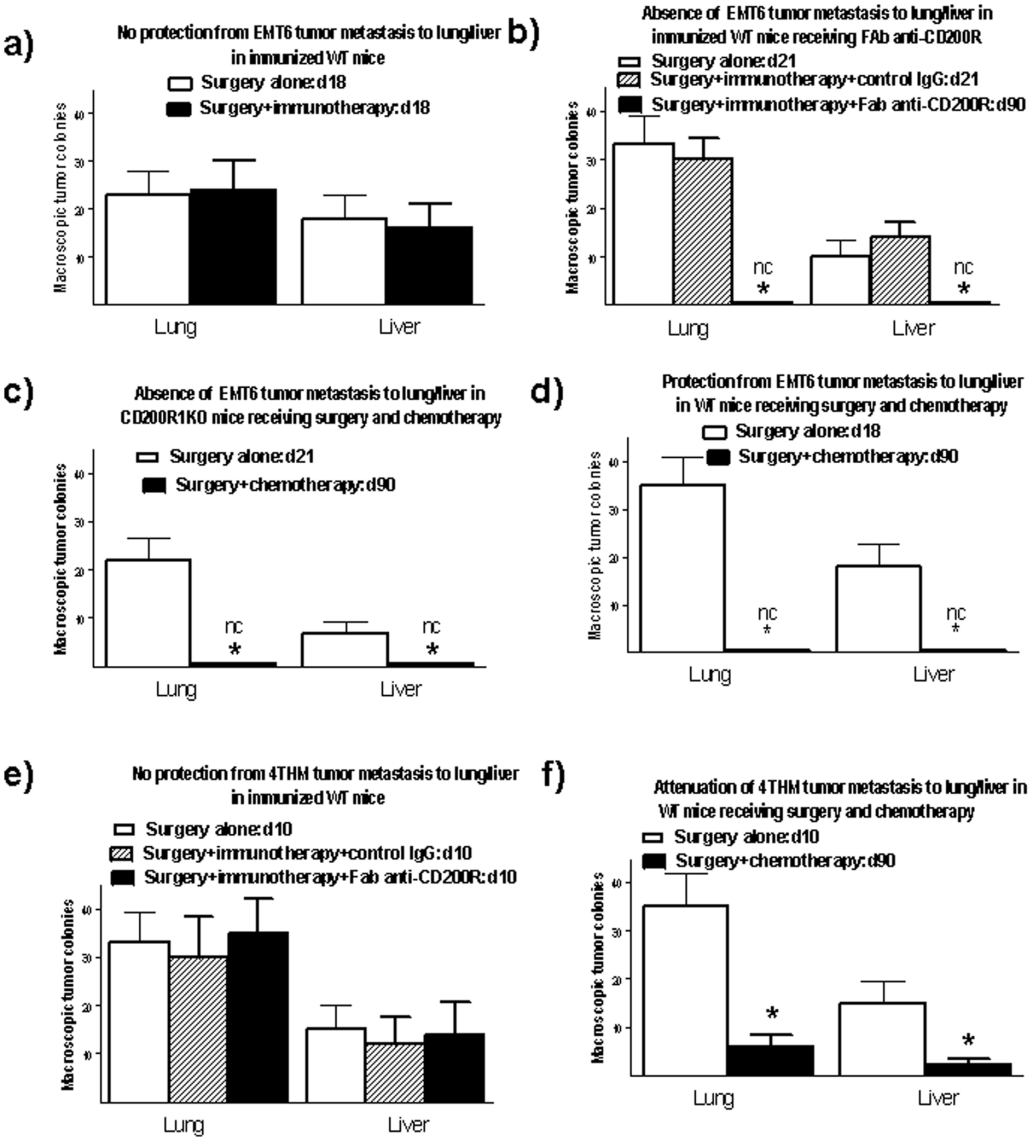
Comparison of lung and liver metastases of tumor cells in WT BALB/c mice receiving EMT6 or 4THM tumor cells and subsequently treated with surgical resection and chemotherapy/immunotherapy (see )Methods. 4 mice were used per group, with mice sacrificed at the times show post surgery (number above histogram bars) to measure macroscopic tumor metastases in the lung/liver. All data represent arithmetic means (±SD) for each group. nc indicates no metastatic colonies detected; *, p<0.05 relative to similar group receiving either immunotherapy or chemotherapy.

Surgical resection of a primary tumor in CD200R1KO mice followed by immunization prevented macroscopic lung/liver metastases enumerated at 90 d post tumor inoculation, compared with surgery alone [9]. As shown in Figure 1 (data pooled from 2 independent studies) no protection was seen in wild type (WT) mice Figure 1, panel a), but WT mice were cured if given Fab anti-CD200R following surgery/immunization (panel b). Note that aggressive tumor growth led to WT control mice having to be sacrificed within 18 d or 21 d of surgery (panels a/b), unlike immunotherapy-treated mice receiving anti-CD200R (panel b) where mice were able to be followed for ≥90 d post surgery. When mice in this latter group were sacrificed earlier (18–21 d post surgery) again no lung/liver colonies were observed (not shown, but note no colonies at 90 d). Both CD200R1KO and WT mice showed no evidence of macroscopic metastases following chemotherapy instead of immunotherapy post surgery (Figure 1, panels c/d respectively). Again note that addition of chemotherapy treatment allowed mice to be monitored for tumor metastases (90 d post surgery) much longer than non-chemotherapy controls (21 and 18 d in panels c, d respectively-however, in studies where chemotherapy mice were deliberately sacrificed early, no metastases were observed on days 18/21 (not shown-but note data for 90 d). In mice receiving 4THM tumors, attenuation of lung/liver metastasis was achieved using surgery+chemotherapy, but not by surgery followed by immunotherapy (see Figure 1, panels e and f respectively). Failure of immunotherapy to protect from 4THM tumors again led to these mice (panel e) being sacrificed much earlier (10 d post surgery) than with EMT6 mice (panels a–d) or 4THM mice receiving chemotherapy (panel f). Once again, in studies where chemotherapy-treated 4THM injected mice were sacrificed at 10 d post surgery, no metastases were seen (not shown-but seen marked attenuation of metastases even at 90 d in panel f).

**Figure 2:**
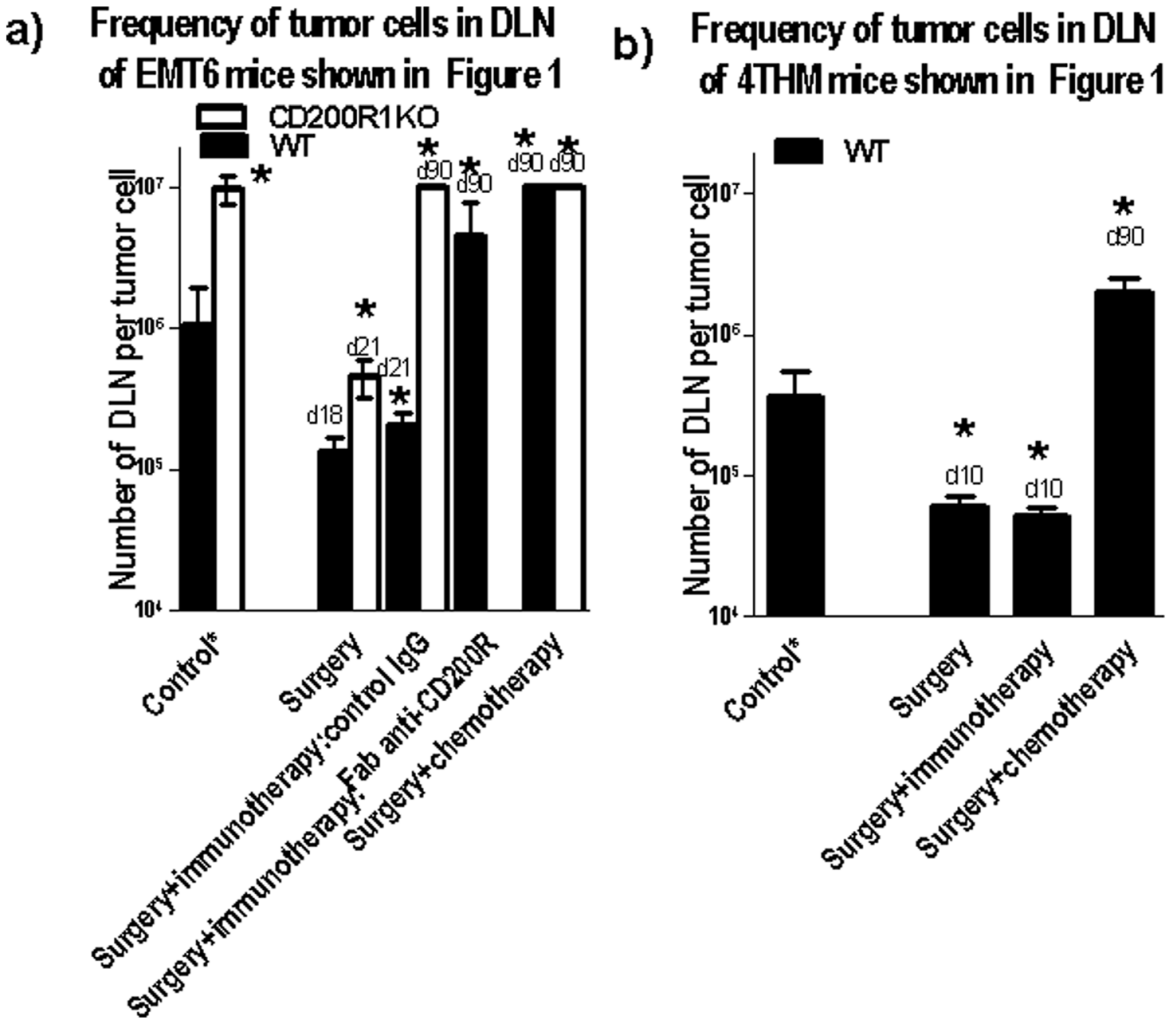
Attenuation of outgrowth of tumor from DLN of mice shown in **Figure 1** as assessed by limiting dilution frequency (see Methods). DLN cells from separate mice were also cloned alone at the time of surgery (data to far left of each panel-control*). All frequencies were calculated based on the input numbers of cells from DLN of control mice only. *, p<0.05 compared with control* mice

DLN cell suspensions of mice sacrificed at the times shown in Figure 1 were cultured under limiting dilution conditions with cultures monitored over a 21-day period for colony growth, to enumerate the frequency of tumor cells in the initial DLN samples (Figure 2: panel a shows data for EMT6 tumors, panel b for 4THM) [7]. Data to the far left in each panel show the frequency of tumor cells cloned from DLN of mice sacrificed on the day of tumor resection. Cells in all clones were stained (∼100% positive) with anti-BTAK (anti-tumor) antibody (data not shown-see [7]).

The frequency of tumor cells cloned from DLN of both WT and CD200R1KO EMT6-injected mice treated only by surgical resection increased over 18–21 d post resection, relative to the frequency seen in DLN at the time of surgical resection (panel a). Surgical resection followed by immunotherapy and control IgG led to little decrease in the DLN tumor frequency in WT mice sacrificed at 21 d post surgery. Fab anti-CD200R along with surgery/immunization resulted in a marked decrease (>7x) in tumor cells cloned from DLN of WT mice (d90). In similarly treated CD200R1KO mice no tumor cells were detected (detection limits in assay ∼1 in 1×10^7^) at 90 d post surgery. No detectable tumor cells could be cloned from DLN of either WT or CD200R1KO mice 90 d post surgery if animals received chemotherapy following surgical resection (data to far right in Figure 2a). In 4THM tumor-bearers (panel b), sacrifice of mice 10 d after surgery with either no additional treatment, or immunotherapy (CpG+ irradiated 4THM), indicated an increase (∼8x) in frequency of cloned tumor cells in DLN compared with the numbers present at the time of surgery. Surgery followed by chemotherapy decreased the number of cloned tumor cells at d90 (far right in Figure 2b).

In separate studies (not shown), no WT or CD200R1KO mice survived following treatment with surgery and anti-VEGF alone, and survival with paclitaxil as the sole chemotherapeutic agent was ≤50% of that seen using the combination shown, in both CD200R1KO and WT mice with each tumor used. Combined surgery and chemotherapy "cured" WT mice of EMT6 tumor growth, as defined by an absence of macroscopic metastases at 300 d post surgery, and undetectable tumor cells cloned from DLN of mice at this time (limits of detection ∼1 in 2×10^7^ DLN cells)-see also [9]. All 4THM mice treated in this fashion died before110days post surgery (data not shown).

### Absence of cells attenuating ability to clone tumor from DLN of mice receiving chemotherapy

Figure S1 investigated whether DLN of either immunotherapy- or chemotherapy-treated WT mice contained populations of cells which non-specifically attenuated growth of tumor cells, leading to inaccurate estimation of tumor cell frequency in limiting dilution [9]. Groups of 5WT mice were treated as in Figure 1 with EMT6 or 4THM tumor cells, followed by surgical resection and combined chemotherapy with anti-VEGF and paclitaxil. Mice were sacrificed 90 days post surgery. DLN cells from WT mice receiving either EMT6 or 4THM tumor cells 14d earlier (WT* in Figure S1) were cultured under limiting dilution conditions (from 2×10^3^ to 1×10^5^ cells/well) alone, or with a five-fold excess of DLN cells from the 90d chemotherapy-treated mice (from 1×10^4^ to 5×10^5^). Cells from these WT or CD200R1KO mice were also cloned alone. All tumor cells frequencies were subsequently calculated based on the input numbers of control cells only. Data shown in this Figure are pooled from 3 separate studies.

The frequency of detected tumor cells in the mice at 90 d post combined surgery/chemotherapy was below the limits of detection in this assay (see data to far right in each of the EMT6/4THM groups of Figure S1). Addition of a 5-fold excess of cells from the DLN of these populations ***did not*** alter the measured frequency of cloneable tumor cells from DLN of WT* mice sacrificed at 14 d post tumor injection.

### CD4^+^ cells in immunotherapy-treated, but not in chemotherapy-treated mice, are responsible for decreased metastasis

Protection (in CD200KO or CD200R1KO mice) was not related to a direct immune response from recipient mice to CD200 expressed on tumor cells themselves [9], [25]. CD200/CD200R is not expressed on 4THM tumors, and thus an immune response to such tumor-bearing epitopes could not explain the differences observed above. Immunotherapy of EMT6 tumor growth was abolished by infusion of anti-CD4 mAb [9]. To investigate whether an active CD4-dependent immune process was implicated in protection afforded by (surgery + chemotherapy) we performed the following study.

**Figure 3:**
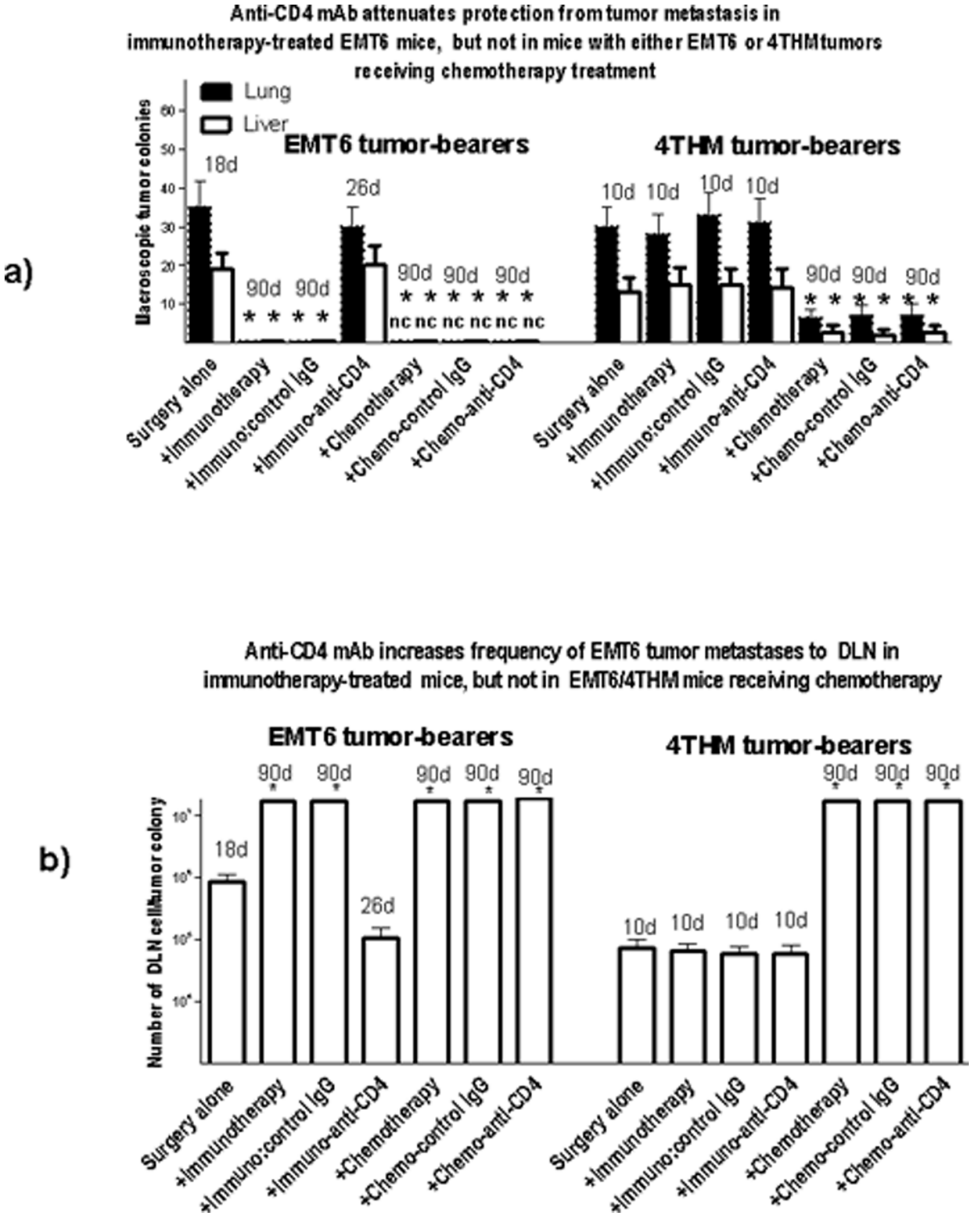
Effect of anti-CD4 mAb on lung/liver (panel a) or DLN (panel b) metastases in mice receiving EMT6 or 4THM tumor cells and treatment as in **Figure 1**. 5 mice were used per group for sacrifice at the time post surgery points shown (numbers above histogram bars). Data show means for macroscopic tumor colonies/group; nc =  no visible tumor colonies. * indicates p<0.05, compared with control treated with surgery alone;

Groups of 30 WT mice received EMT6 or 4THM cells into the mammary fat pad, followed by surgical resection. 5 mice/group received no further treatment. Two subgroups of 15 mice each then received either combination chemotherapy, or immunotherapy with irradiated tumor cells, CpG and Fab anti-CD00R. 10 d after immunotherapy/chemotherapy was initiated 5mice/group began a course of anti-CD4mAb or control IgG injections (3 injections of 75 µg in 300 µlPBS at 72 hr intervals iv). Mice were monitored for overall health, with sacrifice of all mice when there was evidence of respiratory distress and/or weight loss (10%) in any individual. Note that in the case of 4THM mice not receiving chemotherapy, this necessitated sacrifice at 10 d post surgery, while for EMT6 control mice, or EMT6 mice receiving immunotherapy and anti-CD4 treatment, this necessitated sacrifice at18, 26 d post surgery respectively (see also text to Figure 1 above). All surviving mice were terminated at 90 d post surgery, and macroscopic liver/lung metastases determined, along with frequency of tumor cells in DLN (see Figure 2). In addition (see Figure S2), splenocytes from individual mice were stimulated in vitro with irradiated tumor cells for 6 d, with cytokine production measured (48 hr) and CTL assayed at 6 days, as described in the Methods. Data for 1 of 3 such studies are shown in Figure 3.

Macroscopically visible metastases in lung/liver (Figure 3a), along with increased frequency of tumor cells cloned from DLN (Figure 3b), was seen in EMT6 tumor injected mice receiving immunotherapy and anti-CD4 relative to mice receiving control Ig (see also [9])-as noted in Figure 1, where other immunotherapy-treated (but no anti-CD4) EMT6 groups were sacrificed at d18/26 (not 90 d as shown) there were, as expected, no metastases seen. Also as noted in Figure 1, immunotherapy afforded no protection from 4THM growth, regardless of subsequent anti-CD4 treatment, and these mice had to be sacrificed early in the study (10 d post surgery, by comparison to chemotherapy-treated mice, sacrificed at 90 d post surgery). In contrast to these data, following both EMT6 and 4THM tumor injection, the protection from macroscopic (lung/liver) and microscopic (DLN) metastases afforded by chemotherapy was apparently resistant to anti-CD4mAb therapy (Figure 3a/b). In separate studies (not shown) no affect was seen after infusion of anti-CD8 mAb into chemotherapy treated mice either. These in vivo studies need to be seen in the context of data from Figure S2, showing elevated cytotoxicity (CD4^+^-dependent) only using splenocytes from immunotherapy-treated EMT6 tumor-injected mice (panel b), while in turn CD4^+^ cells from these same mice produced increased cytokines (TNFα, IL-2 and IFNγ) relative to mice receiving surgery alone. Note that in the cytotoxicity assay used in Figure S2b, killing itself was a function of CD8^+^ cells in all groups (data not shown).

### Resistance to implantation of fresh EMT6, but not 4THM, tumor in immunotherapy-treated EMT6-injected mice, but not in chemotherapy-treated EMT6/4THM-injected mice

The data in Figure 3 show that cure of both EMT6- and 4THM-injected mice of macroscopic and microscopic (DLN) tumor metastases following surgical resection and chemotherapy is resistant to anti-CD4 treatment, unlike mice cured of EMT6 tumor following surgery and immunotherapy. We next investigated resistance to fresh tumor implants of the same or different tumor in mice cured following immunotherapy/chemotherapy.

**Figure 4:**
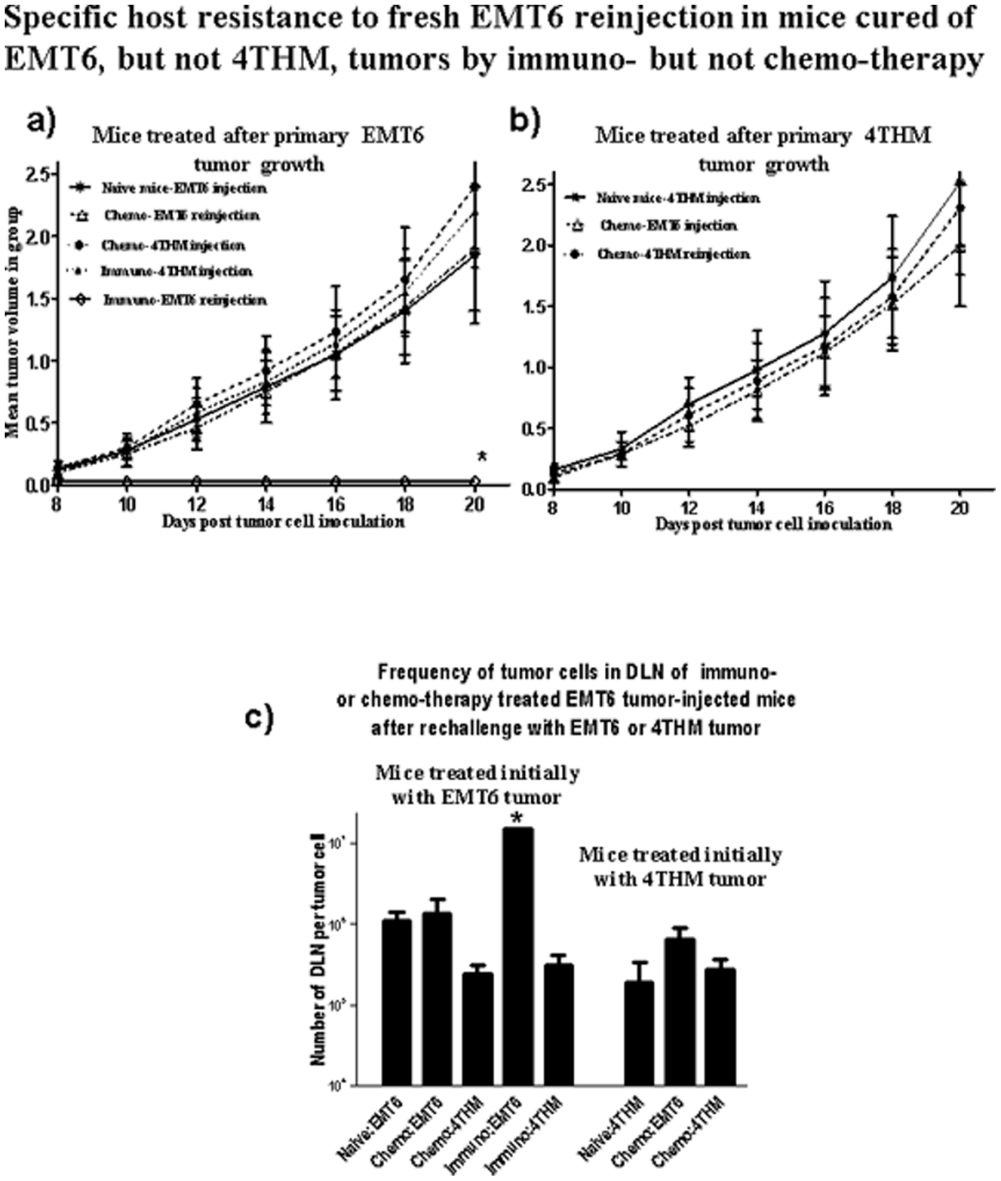
Specific protection from re-challenge with EMT6, but not 4THM, assaying either local tumor growth (panel a) or DLN metastases (panel c) in mice treated 90 d earlier by surgical tumor resection and immunotherapy. Naïve mice had had no previous EMT6 or 4THM tumor implants. All mice were sacrificed at 20 d post re-challenge. Data represent means for group. No protection was seen in mice initially treated with 4THM tumors before treatment/re-challenge (panel b). *, p<0.05 compared with equivalent fresh control mice.

Groups of mice receiving EMT6/4THM tumors underwent surgical resection, followed by either chemotherapy (for all of 15 4THM- and 15 EMT6-injected mice) or immunotherapy (15 EMT6- injected mice). 90 d post surgical resection, with all animals free of obvious tumor growth and gaining weight, 5 mice/group, and 5 fresh mice, received either 5×10^5^ EMT6 or 1×10^5^ 4THM tumors in the contralateral mammary fat pad to that used previously. Primary tumor growth was followed daily for all mice, and animals sacrificed 20 d later, with DLN harvested to assess tumor cells by limiting dilution. Data in Figure 4 show results (1 of 2 studies) for this experiment. None of the mice not receiving further tumor inoculation developed overt tumor recurrence in this time-data not shown to retain clarity.

Figure 4a shows that mice which undergo surgical eradication of EMT6, followed by immunotherapy, are refractory to re-challenge with EMT6 as monitored over 20 d by either visible tumor (panel a) or microscopic DLN metastases (panel b). There was no such protection seen if re-challenge was with 4THM tumor cells. Growth of either EMT6 or 4THM in mice receiving EMT6 followed by surgery/chemotherapy was equivalent to that seen in naive mice. Mice receiving primary injections with 4THM, and subsequently treated with chemotherapy, showed no resistance to re-challenge with either EMT6 or 4THM (Figure 4b). These data were mirrored by analysis of tumor cells frequencies in DLN of treated/re-challenged mice (Figure 4c). Only EMT6 tumor bearers cured by immunotherapy showed decreased DLN micro-metastasis after re-challenge with EMT6, but not 4THM, tumors. Note however, that in these mice (and mice cured of 4THM and re-challenged with EMT6) we cannot discern whether tumor cells measured were of EMT6 or 4THM origin.

**Figure 5:**
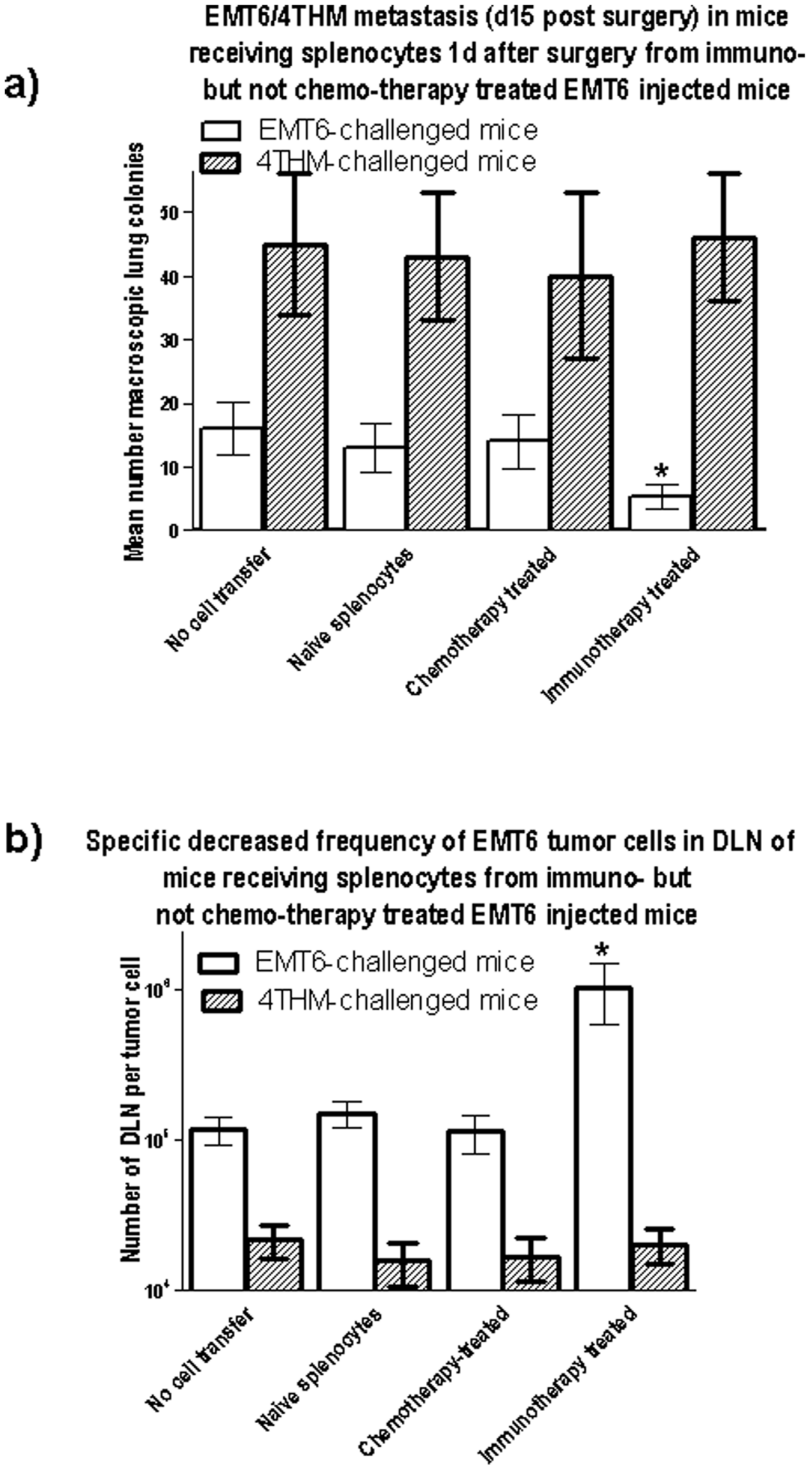
Adoptive transfer of splenocytes from immune- but not chemo-therapy treated mice receiving EMT6 tumors can decrease lung (panel a) and DLN (panel b) metastases in mice which had previously received EMT6 but not 4THM tumors. The tumors in the latter mice were surgically removed 1 d before spleen transfer, and all mice sacrificed 14 d after spleen cell transfer. Data show means (±SD). *, p<0.01 relative to control (no cell transfer).

Further evidence suggesting that immunotherapy, but not chemotherapy, treatment of EMT6-injected mice resulted in protective immunity to re-challenge with the same tumor came from studies using splenocytes pooled from 4mice/group 90 d post either surgical resection of primary tumors followed by either chemotherapy or immunotherapy. 50×10^6^ of these cells were infused iv into fresh mice initially receiving 5×10^5^ EMT6, or 1×10^5^ 4THM, tumor cells (Figure 5) 15 d earlier, and surgically removed 1 d before spleen cell transfer. Lung tumor colonies were enumerated in all groups at 15 days after surgery (14 d after spleen cell transfer), and DLN used to estimate tumor cell frequency by limiting dilution. Data for 1 of 2 studies are shown in Figure 5.

In this independent assay, protection from metastatic tumor colony growth, either macroscopic (to lung) or microscopic (DLN metastases assayed by limiting dilution), was afforded only by transfer of splenocytes from mice cured of EMT6 by surgical resection and immunotherapy, and not from mice cured by chemotherapy. Furthermore, no protection from growth of 4THM tumors was observed.

## Discussion

Breast cancer cells are thought to be continuously monitored by host resistance mechanisms (immunosurveillance [27]), as evidenced by linkage of MHC expression (Class I) with breast cancer growth [28]–[30], as well as analysis of the role of other immune parameters on disease incidence/progression [31]–[34]. Included amongst such studies are several reporting on the possible importance of regulation of inflammation by T lymphocytes [35]–[37]. Consistent with these concepts, lymphocyte infiltration into breast tumors is correlated with improved overall survival [38], and peripheral blood of breast cancer patients show evidence at both the cellular and humoral level of immunity to antigens (MUC-1 and Her-2/neu) associated with human breast cancer [39], [40]. This in turn is reflected in the moderate success seen using Her-2/neu peptides, and other antigenic moieties, as a cancer vaccine [41], [42]. While there remains controversy concerning whether development of CD4 or CD8 immunity will best predict host-resistance [43], [44], there is also concern that vaccination may augment induction of Tregs to block effective tumor immunity [45], [46]. Compounding the complexity of understanding the role of immunotherapy in breast cancer treatment is the potential effect of concomitant chemotherapy on the immune system of the tumor host. Conventional cyclophosphamide-methotrexate-5-fluorouracil (CMF) chemotherapy decreases both NK cell activity [47]. In contrast, in studies of taxane-based chemotherapy in 30 women with advanced breast cancer, increased NK and LAK cell activity and increased IL-6, GM-CSF, and IFNγ levels with decreased IL-1 and TNFα levels were reported in cancer patients following chemotherapy, and correlated with clinical responses [48]. Similarly, cyclophosphamide which is known to suppress T reg cells, has been incorporated into some vaccine *HER2/neu* vaccine trials [39].

Anti-CD200 mAb protects mice from micro-metastasis of EMT6 to DLN, while EMT6 over-expressing a CD200 transgene, or growing in CD200^tg^ hosts, grew more aggressively and metastasized at higher frequency [7]. CD200RKO mice were more resistant both to primary and metastatic growth of tumor [25]. In CD200R1KO mice cured (tumor-free for >300 d) by surgical tumor resection and immunotherapy, CD4^+^ cells, rather than effector CD8^+^ cells, were critical for protection [9]. Growth and metastasis of a highly aggressive metastatic variant (4THM) of the breast tumor 4T1 was reported to be refractory to attenuation of CD200:CD200R interactions in CD200R1KO mice [8].

The current studies have extended our understanding of host resistance to EMT6 tumors using WT mice as tumor recipients, and, following surgical resection of tumor, by augmenting immunization with tumor cells (with CpG as adjuvant) with infusion of Fab anti-CD200R to block CD200:CD200R interactions. We compared this treatment with a more conventional approach using surgery followed by chemotherapy with anti-VEGF and paclitaxel, and compared results with EMT6 and the less immunogenic tumor, 4THM. 4THM mice were not effectively treated with immunotherapy, as was evident from the different times at which mice were sacrificed to measure tumor metastases endpoints in Figures 1–3. In contrast, chemotherapy was effective for both EMT6 and 4THM tumors, allowing us to study mice up to 90 d post surgery (Figures 1–3). Data in Figures 3–5, show that: (i) cure following chemotherapy in both tumor models is not abolished by anti-CD4 treatment, unlike cure of EMT6 tumors by immunotherapy (Figure 3-see also [9]). Immunotherapy in the EMT6 tumor model led to increased induction of direct killing (by CD8^+^ effector cells) using splenocytes from treated mice, along with increased cytokine production in vitro-both effects were attenuated in mice receiving anti-CD4 treatment in vivo (Figure S2). (ii) following chemotherapy, mice initially cured of either 4THM or EMT6 tumors were not resistant to re-challenge with the same tumor, though immunotherapy of EMT6 tumors afforded resistance to re-challenge with the same tumor, but not with 4THM (Figure 4); and finally, (iii) only splenocytes from immuno- but not chemo-therapy treated EMT6 mice, could adoptively transfer protection from macroscopic/microscopic metastases to surgically treated WT mice (Figure 5) previously injected with the same tumor. Again no protection was afforded against 4THM tumors. Thus we were able to induce a tumor-protective immune response in WT mice with EMT6 tumors, but not mice with the more aggressive 4THM tumors. Additional features differentiating host inflammatory responses to EMT6 and 4THM have been described elsewhere by Erin et al (8). Given that the sensitivity of detection of metastases from DLN in our limiting dilution assay is ∼1∶10^7^ cells, and that anti-CD4 treatment of immunotherapy-treated EMT6 tumor injected mice reveals increased metastases in mice otherwise “cured” of disease, we speculate that such mice may harbor quiescent tumor cells, whose growth is held in check by mechanisms which are CD4-dependent.

The nature of the resistant mechanism(s) in mice undergoing chemotherapy in the regimen prescribed is not yet clear. Preliminary data show a difference in intra-tumoral cytokine profiles in such animals, and a difference in phenotype of cells infiltrating the re-challenged EMT6 tumor in WT mice compared with those infiltrating a primary tumor challenge, with increased CD4^+^ cells. This in itself is of interest given the data of Figure S2a, showing a CD4^+^-dependent augmented cytokine production (TNFα, IL-2 and IFNγ) in mice receiving immunotherapy, but not chemotherapy. Infusion of exogenous soluble CD200 into mice undergoing chemotherapy treatment did not attenuate cure or increase metastasis (RMG-unpublished), confirming the independence of this protection from an effect mediated by CD200:CD200R interactions, which is clearly implicated in the immunotherapy described. Our data suggest that optimal treatment of breast cancer should take into consideration the importance in "trade-off" between cancer cell sterilization by immunosuppressive drug treatment and the potential benefit of enhancing immune resistance by manipulation of co-inhibitory (CD200) pathways.

## Supporting Information

Figure S1 **DLN cell from (surgery+chemotherapy) treated WT mice do not antagonize outgrowth of tumor clones from DLN of WT mice sacrificed 14d post EMT6/4THM tumor cell injection.** DLN cells from 5/group WT mice were harvested at 90 d post tumor resection and chemotherapy treatment (see Figures 1 and 2), and from separate groups of WT mice 14 d post EMT6/4THM injection-WT* in Figure). Cells from the latter were cultured under limiting dilution conditions (from 2×10^3^ to 1×10^5^ cells/well) alone, or with a 5-fold excess of cells from the 90 d treated mice. DLN cells from the latter were also cloned alone (data to far left in each subgroup in the Figure). All tumor cell frequencies cloned were calculated based on the input numbers of cells from DLN of WT* only.
(TIF)

Figure S2 **Cytokine production (panel a) and CD8^+^-dependent antigen specific lyses of ^3^HTdR tumor target cells (panel b), using splenocytes from mice described in** **Figure 3** **.** Control mice in each panel received no tumor cells-in this case only data are pooled for groups stimulated with either EMT6 or 4THM cells. Other mice shown were injected with EMT6 (left side of each panel) or 4THM tumor (right side of each panel), and received surgery alone, or followed by chemotherapy/immunotherapy. For all these studies splenocytes were harvested at 90 d post surgery, or earlier as necessary for groups where tumor growth was not controlled (see Figure 3), and re-stimulated in vitro with the same tumor cells (EMT6 or 4THM). Data show mean (±SD) for triplicate cultures, with a minimum of 4 individual spleen cells assayed/group. * p<0.05 compared with a surgery-only control group.
(TIF)
